# Application of an ex-vivo drug sensitivity platform towards achieving complete remission in a refractory T-cell lymphoma

**DOI:** 10.1038/s41408-020-0276-7

**Published:** 2020-01-27

**Authors:** Sanjay de Mel, Masturah B. M. Rashid, Xi Yun Zhang, Jasmine Goh, Chun Tsu Lee, Li Mei Poon, Esther H. L. Chan, Xin Liu, Wee Joo Chng, Yen Lin Chee, Joanne Lee, Yi Ching Yuen, Jing Quan Lim, Burton K. H. Chia, Yurike Laurensia, DaChuan Huang, Wan Lu Pang, Daryl Ming Zhe Cheah, Esther Kam Yin Wong, Choon Kiat Ong, Tiffany Tang, Soon Thye Lim, Siok Bian Ng, Soo Yong Tan, Hoi-Yin Loi, Lip Kun Tan, Edward K. Chow, Anand D. Jeyasekharan

**Affiliations:** 1 Department of Haematology-Oncology, National University Hospital, Singapore, Singapore; 2 KYAN Therapeutics, Singapore, Singapore; 3 Cancer Science Institute of Singapore, Yong Loo Lin School of Medicine, National University of Singapore, Singapore, Singapore; 4 Division of Cellular and Molecular Research, National Cancer Centre Singapore, Singapore, Singapore; 5 Division of Medical Oncology, National Cancer Centre Singapore, Singapore, Singapore; 6 Department of Pathology, Yong Loo Lin School of Medicine, National University of Singapore, Singapore, Singapore; 7 Department of Pathology, National University Hospital, Singapore, Singapore; 8 Department of Diagnostic Imaging, National University Hospital Singapore, Singapore, Singapore; 9 Department of Pharmacology, Yong Loo Lin School of Medicine, National University of Singapore, Singapore, Singapore; 10 N.1 Institute for Health, National University of Singapore, Singapore, Singapore

**Keywords:** Translational research, T-cell lymphoma

Dear Editor,

Currently, there are no clinically approved methods for predicting the relative efficacy of drug combinations for individual patients with cancer. Ex-vivo drug sensitivity experiments with patient-derived tumor material potentially offers a solution to identifying appropriate combinations of therapies for individual patients^1^. However, such assays are typically limited by the quantity available for combinatorial analysis of multiple drugs. We recently developed an experimental–analytical hybrid method, Quadratic phenotypic optimization platform (QPOP), which ranks drug combinations using a limited amount of tumor^2^. QPOP identifies optimal combinations based on the observation that biological response to perturbations (such as therapeutic intervention) can be mapped to a second-order polynomial equation^3^. In our initial study, QPOP identified novel therapeutic combinations for drug-resistant multiple myeloma, using ex-vivo testing on primary tumor samples. However, the concordance of QPOP-based drug sensitivity prediction with actual patient response to treatment was not explored in that study. We now present a case illustrating the setup and utilization of a QPOP protocol as a clinical decision aid, to identify an optimal salvage regimen for a patient with refractory lymphoma.

A 55-year-old male presented to the Hematology Department at the National University Hospital (NUH) Singapore for a second opinion with a refractory lymphoma. He was initially diagnosed and treated in his home country for NK/T-cell lymphoma/leukemia with SMILE (steroid, methotrexate, L-asparaginase, etoposide) and subsequently GDP (gemcitabine, cisplatin, and dexamethasone). His disease progressed rapidly after both these regimens, prompting him to travel to our center for further evaluation. On presentation at NUH, the patient had weight loss, massive splenomegaly, anemia (hemoglobin 8.6 g/dl), thrombocytopenia (25 × 10^9^/L), and lymphocytosis (absolute lymphocyte count 65 × 10^9^/L). A computed tomography (CT) scan revealed massive splenomegaly with no evidence of lymphadenopathy elsewhere. A bone marrow aspirate (BMA) showed infiltration with atypical lymphoid cells comprising 89% of nucleated cells. Flow cytometry (FC) immunophenotyping of the malignant cells identified an aberrant CD3−/CD7+/CD8dim/CD4−/CD16+/CD56+/CD2heterogeneous phenotype. The bone marrow trephine biopsy showed a lymphoid infiltrate featuring small lymphocytes with condensed chromatin and inconspicuous nucleoli with a sinusoidal pattern of infiltration (Fig. S1). Immunohistochemistry (IHC) showed positive expression of CD2 and CD7 (Fig. S1), but negativity for CD3 and Epstein Barr Virus encoded RNA. CD56 was weakly positive while T-cell receptor (TCR) gamma was expressed. Based on the clinical features, bone marrow morphology, IHC and FC phenotype, the diagnosis was revised to Hepatosplenic T-cell Lymphoma (HSTCL). HSTCL is a rare subtype of peripheral T-cell lymphoma (PTCL) with gamma-delta neoplastic T-cells, and a typical clinical presentation of hepatosplenomegaly, B-symptoms, and cytopenias due to bone marrow (BM) involvement^4^. Patients with HSTCL have an extremely poor outcome, with five-year survival rates of less than 10%^5^.

He was initially treated at our center with the B arm of the HyperCVAD regimen, which the disease was refractory to. This was followed by treatments with pembrolizumab, GVD (gemcitabine, vinorelbine, liposomal doxorubicin) and pralatrexate. All of these had virtually no effect on his disease, and his peripheral white blood count rose to 200 × 10^9^/L, with worsening splenomegaly and B-symptoms. During this period, the patient was also recruited onto a translational research protocol; domain specific review board (DSRB) 2017/00507, for the development of ex-vivo drug sensitivity testing in lymphoma. Details of the protocol and methods are described in the supplementary information. Briefly, in this case, the tumor sample was collected using standard venesection, and mononuclear cells were isolated from 10 ml of a patient blood sample for short-term culturing of primary HSTCL. Combinatorial drug sensitivity analysis was performed by the QPOP method (Fig. S2), where the drug candidates comprised of standard regimens and active agents for T-cell lymphoma, chosen in consideration of the treatment history of the patient (Table S1). An orthogonal array composite design (OACD) was used to minimize the number of combinations for factor screening and in-depth analyses, in concentrations that represent clinically approved doses^6^. After 48-h drug treatment, the CellTiter-Glo® Luminescent Cell Viability Assay was used to quantify cell viability.

Eleven drugs at three doses were used in the initial QPOP experiment on the patient’s sample. All possible permutations of these drugs were ranked based on a coefficient-specific quadratic function. Most conventional drug combinations ranked relatively low in the QPOP assay. For example; SMILE was ranked 3951 out of 14,784 possible 5-drug combinations, while GDP was ranked 493 out of 1320 possible 3-drug combinations (Tables S2 and S3, Fig. S3). Among all possible 3-drug sets, a combination of Cisplatin, Cytarabine, and L-asparaginase was predicted to be most efficacious for this patient’s sample, which could potentially indicate utility of the DECAL regimen^7^. This pediatric regimen, however, is not a standard adult treatment protocol. Ex vivo sensitivity to pralatrexate as a single-drug was also evaluated by dose-response analysis, as the patient was planned for treatment with pralatrexate at the time of sample collection. The patient sample was largely unresponsive to pralatrexate (Fig. 1a). The in-vitro analyses of GDP and pralatrexate were concordant with the refractory responses observed in the patient following treatment with these regimens.

**Fig. 1:**
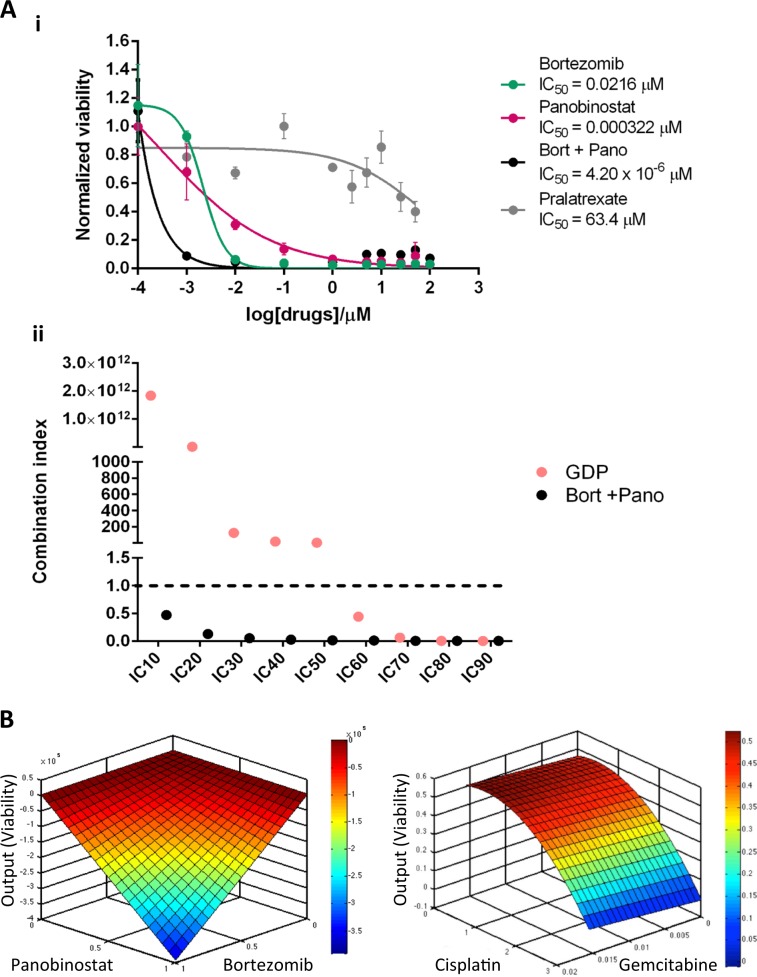
Ex vivo drug sensitivity results. Ex vivo synergistic interaction of combinatorial therapy BP, compared to chemotherapy (**a**(**i**)) dose-response curve of bortezomib, panobinostat and pralatrexate as single agents, and the BP combination. The IC_50_s were 0.0216 μM, 0.000322 μM and 63.4 μM for single agent bortezomib, panobinostat, and pralatrexate respectively. (**a**(**ii**)) combination indices of BP in relation to GDP. Data are presented as means ± SD of three technical replicates. **b** Response surface maps illustrating the interaction between BP (left panel) and Gem-Cis (right panel).

However, we noted a significant efficacy for bortezomib as monotherapy in the sample, highlighting the ability of QPOP to identify both mono- and combinatorial therapy from a single experiment. The dual treatment of bortezomib and a histone deacetylase inhibitor, panobinostat, was previously reported to be efficacious in a Phase II trial on relapsed or refractory PTCL patients, although HSTCL cases were not included^8^. Therefore, a dose-response analysis was performed on the patient sample for bortezomib and panobinostat, in isolation and in-combination (Fig. 1a). There was a significant shift in the dose-response curves when the drugs were administered in combination (Fig. 1a(i)), with combination indices of <1 across different concentrations: indicative of a synergistic interaction (Fig. 1a(ii)). Integrating this serial dose-response dataset into QPOP analytics, we cross-compared the bortezomib-panobinostat combination with the predicted output of other regimens from the initial analysis (Fig. 1b). Response surface maps of bortezomib and panobinostat revealed a sharp decrease in viability as the concentrations increase, suggesting a highly synergistic interaction between bortezomib and panobinostat. As such, it was predicted that the patient would respond better to a combination of bortezomib-panobinostat (BP) than to the other drug combinations evaluated in the assay. Our protocol allows for the sharing of drug sensitivity analysis with the treating physician, and for subsequent genomic analysis of the tumor (DSRB study 2015/00176).

This patient had personal funds to cover the costs of off-label use of novel anti-cancer agents. Given the exquisite ex-vivo sensitivity noted to the BP combination, the lack of any standard treatment options, and the availability of a protocol for BP as described by Tan et al.^8^, a shared decision was made with the patient to initiate treatment with panobinostat 20 mg three times per week and bortezomib 1.3 mg/m² intravenously twice per week. After two cycles of BP, we saw a dramatic reduction in his lymphocytosis (Fig. 2a) The patient was in complete metabolic remission based on a positron emission tomography scan after 8 cycles of BP. (Fig. 2b, c). A bone marrow study showed no evidence of residual disease by morphology or FC. The patient underwent an autologous stem cell transplant (AutoSCT) as he had no matched donor for allogeneic transplant (AlloSCT) and reported wellness 1 year later. Complete remissions (CR) in HSTCL are rare, even more so after salvage therapy in refractory disease^5^. This represents, therefore, a unique case of HSCTL achieving CR with a bortezomib-panobinostat combination identified through ex-vivo drug sensitivity testing. Bortezomib has shown in vitro synergism with histone deacetylase (HDAC) inhibitors against T-cell lymphoma cells^9,10^. However, molecular markers of BP response are unknown. A comprehensive genomic and transcriptomic analysis was performed and is provided here (Tables S4 and S5, Fig. S4, EGA accession number: EGAD00001005229) to facilitate further studies into the molecular basis of this exceptional response in a typically fatal malignancy.

**Fig. 2:**
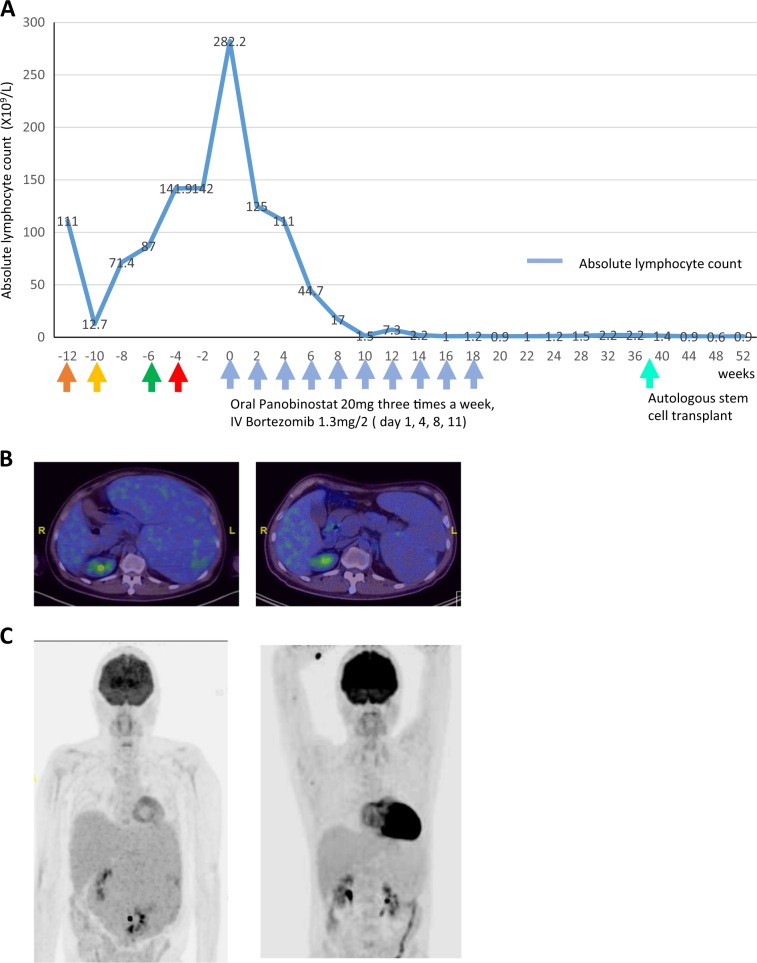
Clinical Response to Treatment with BP. **a** The trend of the absolute lymphocyte count after treatment with each regimen. Color-coded arrows indicate treatment regimens given before bortezomib-panobinostat (blue arrows). Orange: HyperCVAD B, yellow: pembrolizumab, green: gemcitabine, vinorelbine, liposomal doxorubicin, red: pralatrexate. A rapid and sustained reduction of the ALC was seen after treatment with the BP regimen. This was accompanied by an improvement in hemoglobin and platelet count (data not shown). PET scan. **b** The post treatment scan shows an interval significant reduction in size of splenomegaly, measuring 15.6 cm craniocaudally compared with 27.9 cm at initial presentation. **c** The splenic FDG uptake has normalized in the interim. The previous scan shows heterogeneous increased FDG accumulation indicating disease involvement.

Ex-vivo drug sensitivity testing platforms using primary tumor samples hold the promise of identifying appropriate therapies for specific patients^1,11,12^. Previously published platforms rely on comparative single-drug or pairwise-drug sensitivity from multiple tumor samples, sometimes combined with large scale genomic analysis to build pharmacogenomic models^13,14^. We have previously demonstrated that QPOP rationally identifies optimal drug combinations in a sample-specific manner in myeloma cell lines and ex-vivo primary myeloma cells^15^. By applying a QPOP-based protocol towards ex-vivo drug combination sensitivity analysis of primary HSTCL cells from the patient described in this paper, an actionable drug combination in bortezomib and panobinostat was identified within one week of sample collection. Specific drug panels will need to be tested in a larger number of cases to validate the relationship between in-vitro and in-vivo potency of QPOP identified combinations. Ex-vivo strategies have limitations in that tumor cell kill is assessed in the absence of the microenvironment and at doses that may not be accurately reflective of in-vivo levels. However, the high concordance between the QPOP assay and the clinical response in our index case highlights the feasibility and rationale for prospective studies and clinical trials investigating this platform in relapsed/refractory lymphoma.

## Supplementary information

Supplementary material
