# Supplementary material for: Application of an ex-vivo drug sensitivity platform towards achieving complete remission in a refractory T-cell lymphoma
Source: Blood Cancer J. 2020 Jan 27;10(1):9. doi: 10.1038/s41408-020-0276-7 (PMC6985240; doi:10.1038/s41408-020-0276-7)
Supplement: Supplementary file 1 — Supplementary material [file 41408_2020_276_MOESM1_ESM.docx]

**Supplementary information:**

## Tissue and clinical data collection:

We designed a translational research protocol; domain specific review board (DSRB) 2017/00507, for the development of ex-vivo drug sensitivity testing in lymphoma. Blood samples from cases were collected using standard venesection methods. Informed consent was obtained for the procedures described in this manuscript, as per the approved consent forms 2017/00507 and 2015/00176. Clinical data were extracted from electronic medical records at the National University Cancer Institute Singapore. Histopathology images were obtained from representative bone marrow slides, using a Leica microscope at 40x. Representative radiology images were obtained from PET scans using the Siemens Biograph mCT 64 slices PET/CT scanner. Images were acquired from the vertex of the skull to the mid-thigh 60 minutes after intravenous administration of 18F-Fluorodexoyglucose (FDG).


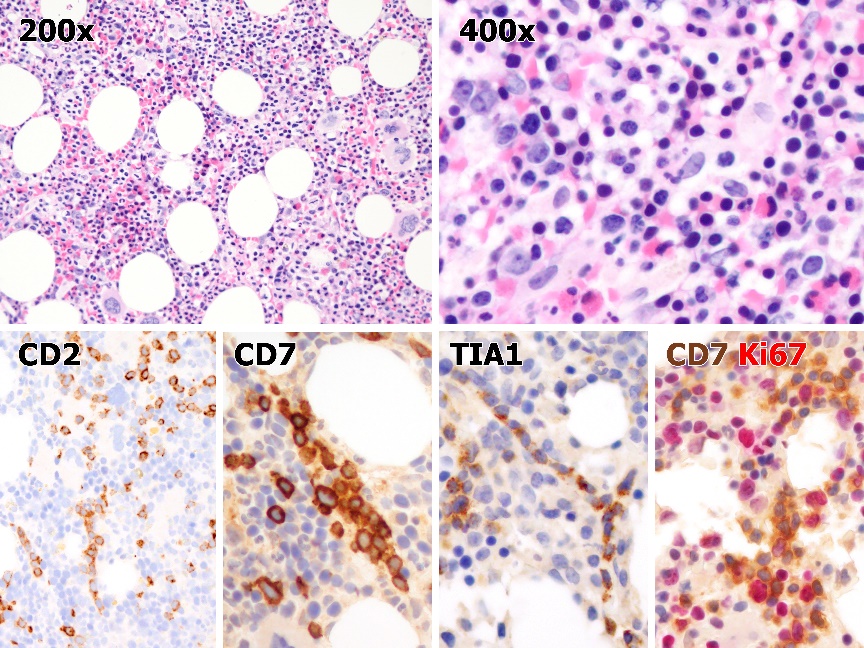

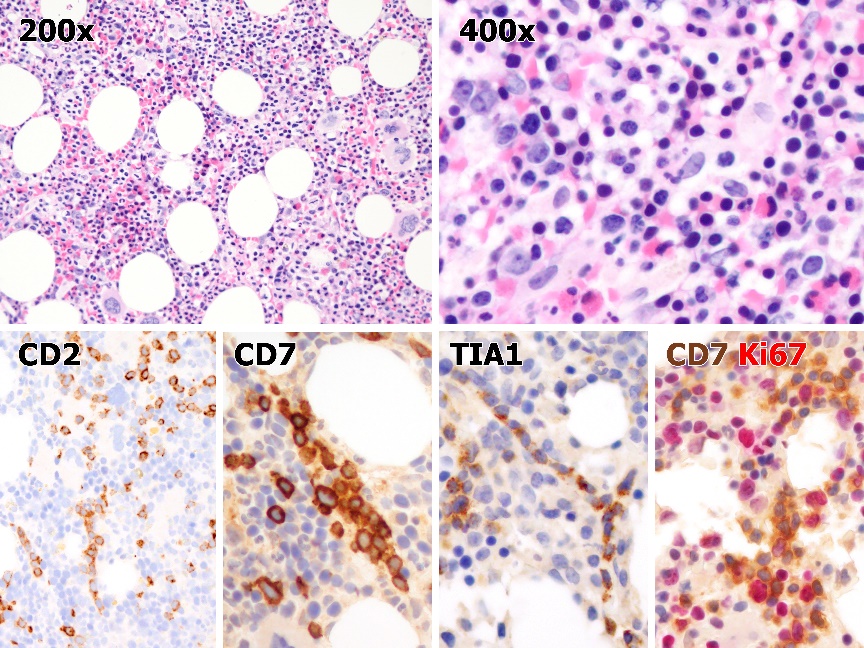


Figure S1. BM trephine. The lymphomatous infiltrate is not discernible with H/E staining at both 200x and 400x magnifications. Immunostains highlight the linear aggregates of CD2+ CD7+ cytotoxic T-lymphocytes, in keeping with sinusoidal pattern of infiltration.

**Ex-vivo drug combination sensitivity analysis:**

Upon collection of the tumor sample, the specimen was transferred on ice to the laboratory in RPMI medium within a 2-hour time period. For short-term culturing of primary HSTCL, mononuclear cells were isolated from 10 ml of patient blood sample using Ficoll-Paque™ (GE Healthcare) and cultured in RPMI-1640 medium (Sigma) supplemented with 10% of fetal bovine serum (FBS; Sigma), 1% of Pen-Strep (Gibco) for subsequent experiments. A trypan blue staining was performed prior to seeding of cells for experimentation, to ensure viability.

Cells were seeded 2000/well in Nunc™ 384-Well Clear Polystyrene Plates. Cell viability was performed using the CellTiter-Glo® Luminescent Cell Viability Assay following the manufacturer's instructions (Promega, Madison, WI, USA). For dose-response assays, multiple concentrations of pralatrexate, bortezomib, panobinostat, gemcitabine, dexamethasone and cisplatin were tested as either single or combinatorial treatments (100, 50, 25, 10, 5, 1, 0.1, 0.01, 0.001, 0.0001μM). The IC_50_ values of the different compounds were determined using non-linear regression plots with the GraphPad Prism v5 software.

**Quadratic Phenotypic Optimization Protocol (QPOP)**

Single patient *ex vivo* drug sensitivity predictors typically require large amounts of tumor sample to test adequate combinations to be of clinical use. QPOP was developed to improve on existing systems, based on the concept that quantifiable phenotypic drug dose-responses can be determined by a second-order algebraic equation. As such, patient sample responses to combinations of drugs that fit within either an orthogonal array composite design or serial dose-response assay can be used to derive patient-specific coefficients. These values describe tumor cell response to all potential drug combinations exclusively based on experimentally derived data, without a priori assumptions of mechanism.

QPOP was utilized to identify an optimal clinically-applicable combinatorial drug regimen for this patient, and dose-response drug sensitivity assays were run separately on the same sample to validate QPOP findings.


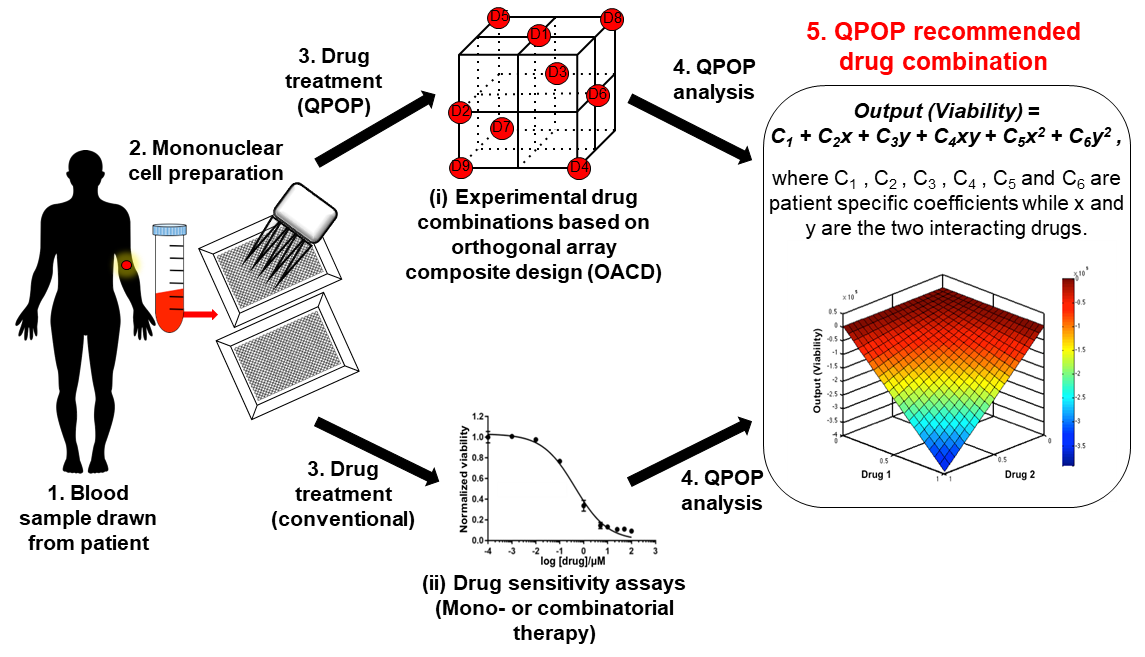


**Figure S2. Overview of the QPOP process.** Blood is drawn from patient (Step 1) and the mononuclear cells are isolated by centrifugation via Ficoll-paque separation. The cells are plated in 384-well plates (Step 2) before undergoing drug treatment (Step 3). This comprises of either a QPOP-specific drug combinatorial treatment (Step 3i) or the traditional serial dose response assays (Step 3ii). The data from steps 3i and 3ii are subjected to QPOP analyses (Step 4). Based on the coefficients derived from the QPOP analyses, QPOP highlights the most optimal patient-specific drug combinations and projects the 2-drug interactions via response surface maps, which aids in clinicians’ decision-making.

**QPOP analytics:**

The drug candidates for the initial experiment comprised of standard regimens and active agents for T-cell lymphoma, chosen in consideration of the treatment history of the patient. The assay was designed from the orthogonal array composite design (OACD) to use the least number of combinations sufficient for factor screening and in-depth analyses[^17^](#_ENREF_17), in concentrations that represent clinically approved doses. After 48-hour drug treatment, the CellTiter-Glo® Luminescent Cell Viability Assay was used to quantify the cell viability.

We carried out an 11-drug, 3- dose level QPOP analysis consisting of 155 combinations, where the 3 levels corresponded to concentrations of the drugs at their IC_0_, IC_15_ and IC_30_ (**Table S1**). All possible permutations of these combinations and their corresponding concentrations were ranked based on a coefficient-specific quadratic function. We sieved out and compared the rankings of conventional drug regimens that the patient had previously received, and others that could potentially be used. These included SMILE (dexamethasone, methotrexate, ifosfamide, L-asparaginase and etoposide), GDP (gemcitabine, dexamethasone and cisplatin), CHEOP (cyclophosphamide, doxorubicin, etoposide, vincristine and prednisone) and ESHAP (etoposide, methylprednisolone, cytarabine and cisplatin). Due to the omission of vincristine, prednisone and Methylprednisolone from the drug set for space constraints, the rankings of CHEOP and ESHAP may not be fully representative of the combinations.


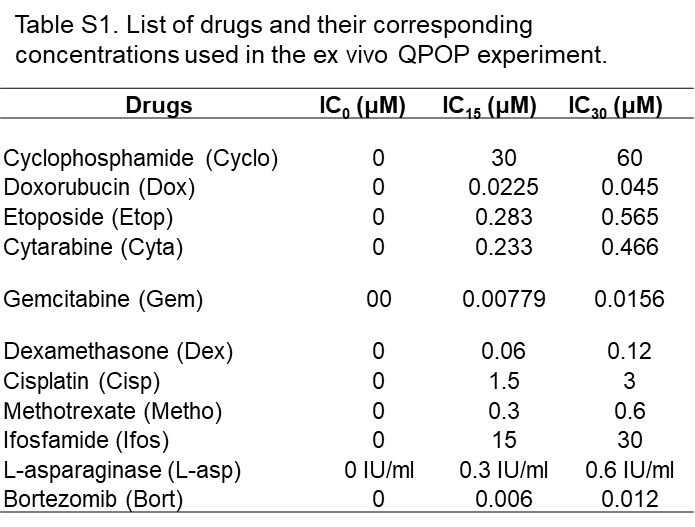


The viability of cells exposed to the various drug combinations are the phenotypic output which is analyzed by the QPOP assay. The correlation of treatment combination (input) and viability (output) was fitted into a second-order quadratic equation, whose coefficients were calculated by MATLAB software (MathWorks Inc.). The coefficients of the second-order equation represent the correlation between the input, drug dose of the drug combination, and the output cell viability, allowing for optimizing drug combination. In the analysis, each drug combination was represented as a vector and coded dosages were used in MATLAB. The second-order quadratic equation is as follows:

y=β_0_ +β_1_x_1_ + ... + β_n_x_n_ + β_12_x_1_x_2_ + ... +β_mn_x_m_x_n_ +β_11_x_1_^2^ + ... +β_nn_x_n_^2^

where y represents the desired output, xn is the nth drug dosage, β0 is the intercept term, βn is the single-drug coefficient of the nth drug, βmn is the interaction coefficient between the mth and nth drugs, and βnn is the quadratic coefficient for the nth drug. Based on the predictive outcomes, drug-drug interactions as well as ranked lists of two drug combinations and three-drug combinations could be derived. To further validate and optimize the top-ranked drug combination, a second-order quadratic fit for each drug combination was made. The coefficients were calibrated using the data points in dose response assay of single drugs and drug combination. To ensure the robustness and integrity of the assay, Z’ score is calculated after every QPOP analysis. It is calculated as follows:$Z=1- \frac{3SD of positive control + 3 SD of negative control}{|mean of positive control - mean of negative control|}$, where SD is the standard deviation between the technical replicates. A score between 0.5 and 1 is indicative of an excellent assay.

**Statistical analysis**

All experiments were performed in at least duplicate technical repeats, unless otherwise stated, with data presented as means ± standard deviation (SD). All experiments were performed in at least duplicate technical repeats, unless otherwise stated, with data presented as means ± standard deviation (SD). Student’s two-tailed t test was used for the comparison of two independent groups.

**QPOP results:**

Table S2. Top 10 5-drug ranked combinations. SMILE listed for comparison


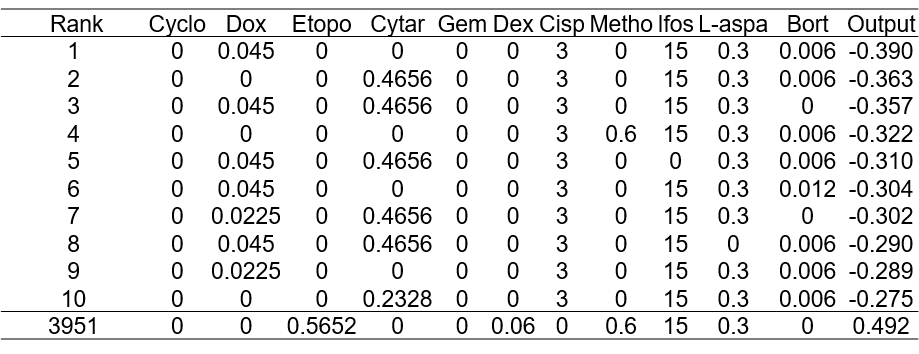


SMILE

Table S3. Top 10 3-drug ranked combinations. GDP listed for comparison


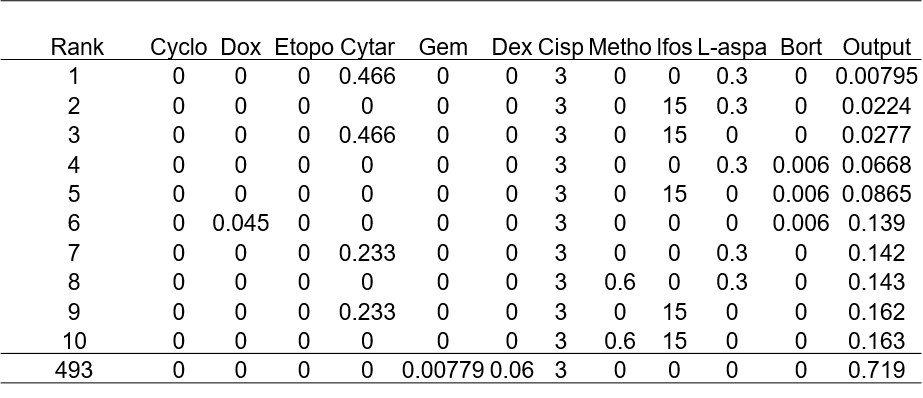


GDP

**QPOP validation:**

For the initial QPOP analysis, we used data from an orthogonal array composite design, with a minimum number of combinations to accurately capture the drug dosing space. Validation of these QPOP findings on conventional chemotherapeutic regimen were performed using dose-response drug sensitivity assays.


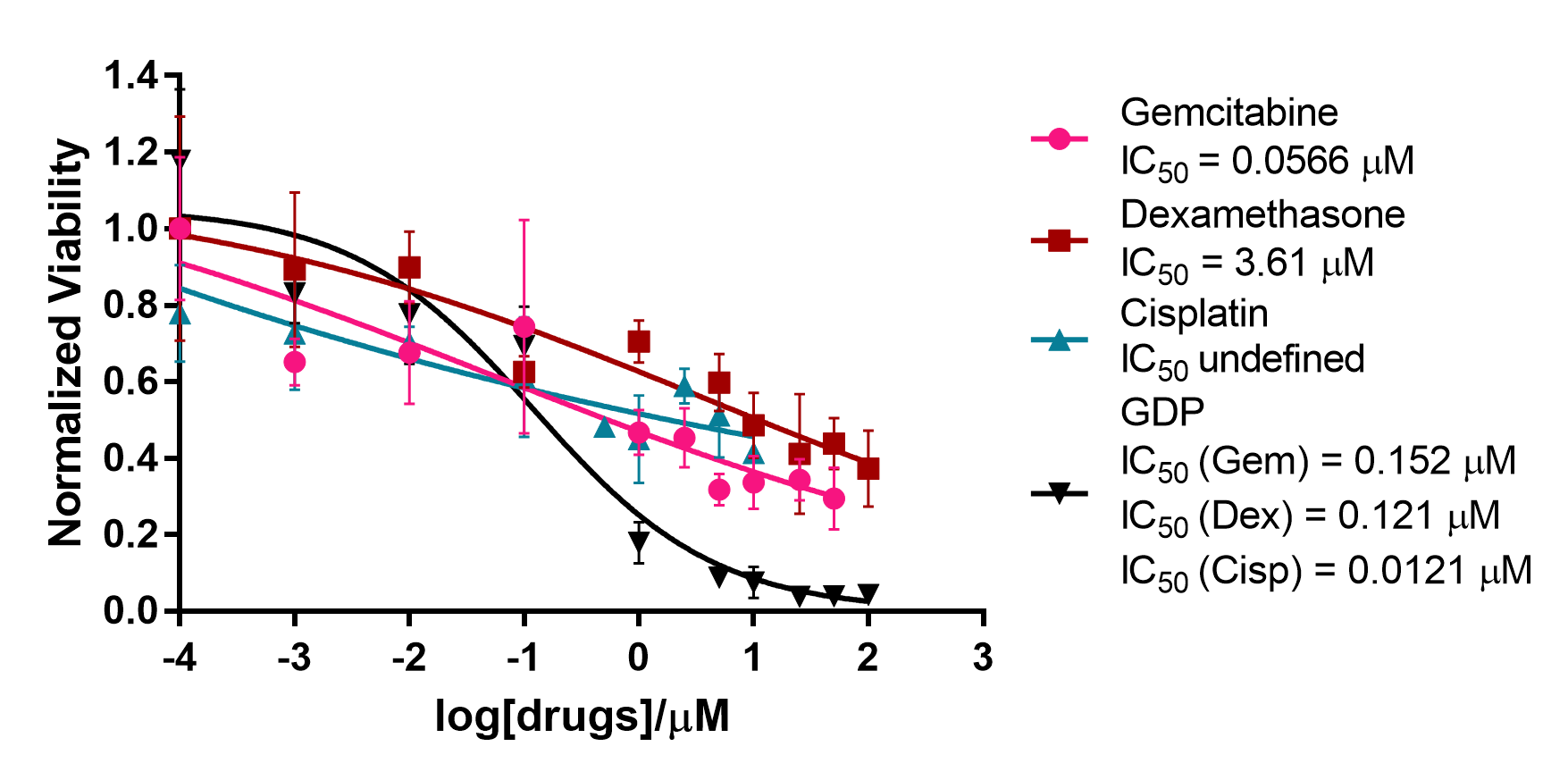


**A**

**B**

**C**


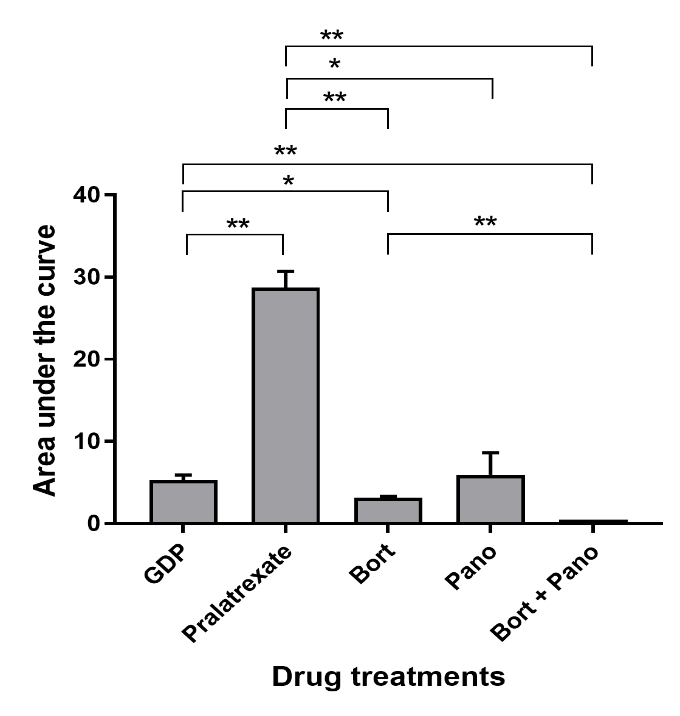


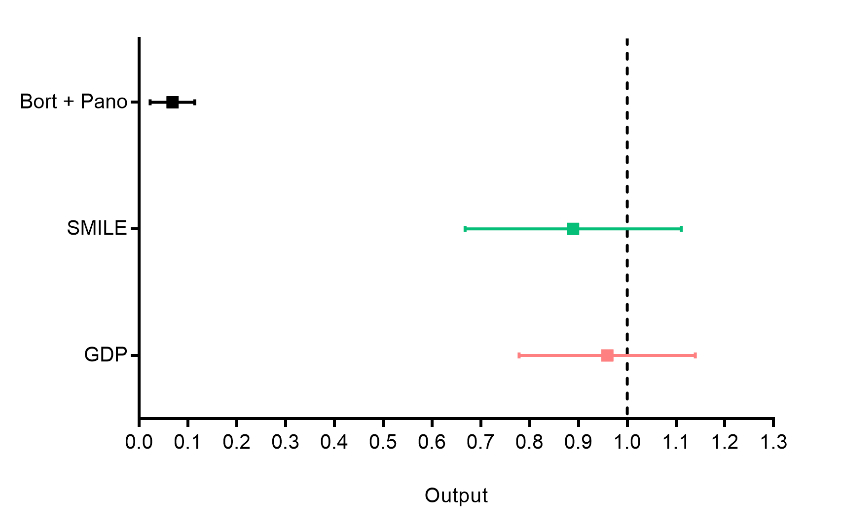


**Figure S3. QPOP results.** A) *Ex vivo* dose-response analysis of the GDP combination compared to single-drug response confirmed the overall antagonistic interactions within this regimen (**Figure S3A**). B) Normalized cell viability (QPOP output) represented between 0 and 1, to compare the relative efficacy of BP in comparison to SMILE and GDP. C) Graphical comparison of the area under the dose response curves for Panobinostat and Bortezomib, for mono- and combinatorial therapy, against GDP and Pralatrexate. All bar plots represent means ± SD, where *P < 0.05 and **P < 0.01. Statistical analyses were performed using two-tailed Student’s t test.

**Genomic and Transcriptomic Analysis**

Peripheral blood and buccal swab were obtained from the patient as approved in the study protocol. Tumor cells were isolated from peripheral blood mononuclear cells by depletion of CD56 negative cells using a human NK cell isolation kit (Miltenyi Biotec), due to the CD56 positive nature of this tumor. Purity of tumor cells was evaluated by CD56-PE staining and samples with >90% CD56+ cells were used. Genomic DNA from tumor cells were extracted using QIAamp DNA Mini kit (Qiagen). For the buccal swab sample, DNA was extracted using EZNA Tissue DNA kit (Omega Bio-tek). Genomic DNA yield and quality were determined by Nanodrop 1000 Spectrophotometer (Thermo Scientific) and Quant-iT Picogreen dsDNA Assay kit (Invitrogen), and visually inspected by agarose gel electrophoresis.

The tumor and normal DNA from this patient were prepared with Illumina Truseq Nano DNA Library Prep kit and sequencing was performed on Hiseq X platform (Illumina). Data was aligned to the hs37d5 reference genome with BWA MEM. The coverage yielded from the alignment from the tumor and buccal swab on the reference genome are 76.7X and 48.3X, respectively. Short-variant calling was performed by Strelka (v1.0.14) and annotated by wAnnovar (as of Jan 2018). Structural variations (SVs) was called by MantaSV (v 0.29.6). Candidate structural variations have to be supported by both spanning and split read-pairs, and have to span at least 1 kb in size. Total RNA from tumor cells was extracted using TRIzol (Invitrogen) and purified with RNeasy Mini kit (Qiagen). The integrity of RNA was determined by electrophoresis using 2100 Bioanalyzer (Agilent Technologies). 1 μg of the total RNA was used for library preparation with Truseq Stranded Total RNA with Ribo Zero (Illumina) and whole transcriptome sequencing was performed on Hiseq 2500 (Illumina) with a total output of 100 million reads. Reads were aligned using STAR (*doi:10.1093/bioinformatics/bts635*) (v2.6.0c) against a combine reference of GRCh37 and EBV in 2-pass mode. The alignment was then quantified at gene level with RSEM (*doi: 10.1186/1471-2105-12-323*) (v1.2.28).

The WGS of the HSTL tumor and matched buccal-swab, as well as the WTS data, are associated with the EGA accession number: EGAD00001005229

To explore the molecular basis of this exceptional response in a typically fatal malignancy, a comprehensive genomic and transcriptomic analysis was performed, and is shared in entirety here. Whole genome sequencing was performed on the tumor and matched normal DNA. A total of 112 non-silent protein-coding mutations were called **(Table S4).** Additionally, a total of 18 SVs were curated **(Table S5).**

**Table S4.** Non silent protein coding mutations identified through whole genome sequencing.

| **Chr** | **Start** | **End** | **Ref** | **Alt** | **Gene.refGene** | **variant read depth** | **total depth** | **VAF** | **Func** | **AAChange.refGene** |
| --- | --- | --- | --- | --- | --- | --- | --- | --- | --- | --- |
| 2 | 211179766 | 211179766 | T | - | MYL1 | 34 | 70 | 0.4857143 | frameshift deletion | MYL1:NM_079420:exon1:c.1delA:p.M1Wfs*6 |
| 9 | 140115424 | 140115424 | C | - | RNF208 | 52 | 107 | 0.4859813 | frameshift deletion | RNF208:NM_031297:exon1:c.241delG:p.D81Tfs*37 |
| 16 | 85689983 | 85689994 | GAGCGCGAGCGC | - | GSE1 | 45 | 97 | 0.4639175 | nonframeshift deletion | GSE1:NM_001134473:exon6:c.712_723del:p.R245_E248del,GSE1:NM_001278184:exon6:c.805_816del:p.R276_E279del,GSE1:NM_014615:exon7:c.1024_1035del:p.R349_E352del |
| 19 | 17948746 | 17948754 | TGCAGTTCT | - | JAK3 | 32 | 83 | 0.3855422 | nonframeshift deletion | JAK3:NM_000215:exon12:c.1688_1696del:p.K563_C565del |
| 22 | 41650469 | 41650471 | TCC | - | RANGAP1 | 5 | 74 | 0.0675676 | nonframeshift deletion | RANGAP1:NM_001317930:exon11:c.1101_1103del:p.E368del,RANGAP1:NM_002883:exon11:c.1101_1103del:p.E368del,RANGAP1:NM_001278651:exon12:c.1101_1103del:p.E368del |
| X | 31089990 | 31089992 | CAG | - | FTHL17 | 4 | 32 | 0.125 | nonframeshift deletion | FTHL17:NM_031894:exon1:c.79_81del:p.L27del |
| X | 31089997 | 31089998 | AT | - | FTHL17 | 4 | 32 | 0.125 | frameshift deletion | FTHL17:NM_031894:exon1:c.73_74del:p.I25Hfs*142 |
| X | 70360680 | 70360682 | GCA | - | MED12 | 4 | 42 | 0.0952381 | nonframeshift deletion | MED12:NM_005120:exon42:c.6240_6242del:p.Q2086del |
| 1 | 1562052 | 1562052 | A | C | MIB2 | 15 | 117 | 0.1282051 | nonsynonymous SNV | MIB2:NM_001170688:exon8:c.A1145C:p.D382A,MIB2:NM_001170689:exon8:c.A800C:p.D267A,MIB2:NM_001170686:exon9:c.A1328C:p.D443A,MIB2:NM_001170687:exon9:c.A1298C:p.D433A,MIB2:NM_080875:exon9:c.A1340C:p.D447A |
| 1 | 90493124 | 90493124 | T | G | ZNF326 | 11 | 49 | 0.2244898 | nonsynonymous SNV | ZNF326:NM_181781:exon10:c.T995G:p.V332G,ZNF326:NM_001320185:exon12:c.T1346G:p.V449G,ZNF326:NM_182976:exon12:c.T1613G:p.V538G |
| 1 | 152080666 | 152080666 | A | C | TCHH | 6 | 94 | 0.0638298 | nonsynonymous SNV | TCHH:NM_007113:exon3:c.T5027G:p.L1676R |
| 1 | 152324599 | 152324599 | A | C | FLG2 | 7 | 64 | 0.109375 | nonsynonymous SNV | FLG2:NM_001014342:exon3:c.T5663G:p.V1888G |
| 1 | 152327578 | 152327578 | C | G | FLG2 | 11 | 92 | 0.1195652 | nonsynonymous SNV | FLG2:NM_001014342:exon3:c.G2684C:p.S895T |
| 1 | 155161762 | 155161762 | T | G | MUC1 | 16 | 110 | 0.1454546 | nonsynonymous SNV | MUC1:NM_001204285:exon2:c.A371C:p.N124T,MUC1:NM_001204286:exon2:c.A398C:p.N133T |
| 1 | 172635031 | 172635031 | C | T | FASLG | 33 | 84 | 0.3928571 | nonsynonymous SNV | FASLG:NM_000639:exon4:c.C721T:p.R241C |
| 1 | 181695211 | 181695211 | A | G | CACNA1E | 32 | 82 | 0.3902439 | nonsynonymous SNV | CACNA1E:NM_000721:exon18:c.A2153G:p.E718G,CACNA1E:NM_001205293:exon18:c.A2153G:p.E718G,CACNA1E:NM_001205294:exon18:c.A2153G:p.E718G |
| 1 | 201180277 | 201180277 | A | G | IGFN1 | 13 | 59 | 0.220339 | nonsynonymous SNV | IGFN1:NM_001164586:exon12:c.A6256G:p.T2086A |
| 1 | 227843321 | 227843321 | A | G | ZNF678 | 4 | 58 | 0.0689655 | nonsynonymous SNV | ZNF678:NM_178549:exon4:c.A1535G:p.E512G |
| 2 | 39029874 | 39029874 | C | T | DHX57 | 4 | 73 | 0.0547945 | nonsynonymous SNV | DHX57:NM_001329963:exon23:c.G3694A:p.V1232I,DHX57:NM_198963:exon23:c.G4000A:p.V1334I |
| 2 | 73678393 | 73678393 | A | T | ALMS1 | 4 | 77 | 0.0519481 | nonsynonymous SNV | ALMS1:NM_015120:exon8:c.A4736T:p.N1579I |
| 2 | 111907656 | 111907656 | C | G | BCL2L11 | 27 | 52 | 0.5192308 | nonsynonymous SNV | BCL2L11:NM_001204106:exon3:c.C160G:p.P54A,BCL2L11:NM_001204107:exon3:c.C160G:p.P54A,BCL2L11:NM_001204108:exon3:c.C430G:p.P144A,BCL2L11:NM_001204110:exon3:c.C160G:p.P54A,BCL2L11:NM_138621:exon3:c.C430G:p.P144A,BCL2L11:NM_138622:exon3:c.C430G:p.P144A,BCL2L11:NM_207003:exon3:c.C160G:p.P54A,BCL2L11:NM_006538:exon4:c.C250G:p.P84A,BCL2L11:NM_138623:exon4:c.C250G:p.P84A |
| 2 | 133542706 | 133542706 | G | A | NCKAP5 | 38 | 80 | 0.475 | stopgain | NCKAP5:NM_207363:exon14:c.C1678T:p.Q560X |
| 2 | 152350292 | 152350292 | G | T | NEB | 35 | 71 | 0.4929578 | nonsynonymous SNV | NEB:NM_004543:exon142:c.C19065A:p.S6355R,NEB:NM_001164507:exon175:c.C24669A:p.S8223R,NEB:NM_001164508:exon175:c.C24669A:p.S8223R,NEB:NM_001271208:exon176:c.C24774A:p.S8258R |
| 2 | 163374597 | 163374597 | C | T | KCNH7 | 37 | 74 | 0.5 | nonsynonymous SNV | KCNH7:NM_033272:exon4:c.G535A:p.D179N,KCNH7:NM_173162:exon4:c.G535A:p.D179N |
| 3 | 47205343 | 47205343 | C | T | SETD2 | 61 | 117 | 0.5213675 | splicing | NM_014159:exon1:c.71+1G>A;NM_001349370:exon1:UTR5 |
| 3 | 138664525 | 138664525 | C | G | FOXL2 | 24 | 63 | 0.3809524 | nonsynonymous SNV | FOXL2:NM_023067:exon1:c.G1040C:p.C347S |
| 3 | 156763171 | 156763171 | C | T | LEKR1 | 37 | 67 | 0.5522388 | nonsynonymous SNV | LEKR1:NM_001004316:exon13:c.C1711T:p.R571C |
| 4 | 2935368 | 2935368 | T | G | MFSD10 | 5 | 94 | 0.0531915 | nonsynonymous SNV | MFSD10:NM_001120:exon2:c.A194C:p.Q65P,MFSD10:NM_001146069:exon3:c.A194C:p.Q65P |
| 4 | 6865435 | 6865435 | G | A | KIAA0232 | 33 | 63 | 0.5238095 | nonsynonymous SNV | KIAA0232:NM_001100590:exon6:c.G3326A:p.R1109H,KIAA0232:NM_014743:exon7:c.G3326A:p.R1109H |
| 4 | 22390094 | 22390094 | G | A | ADGRA3 | 24 | 67 | 0.358209 | nonsynonymous SNV | ADGRA3:NM_145290:exon19:c.C3200T:p.S1067L |
| 4 | 30725796 | 30725796 | G | T | PCDH7 | 32 | 63 | 0.5079365 | nonsynonymous SNV | PCDH7:NM_001173523:exon1:c.G2752T:p.D918Y,PCDH7:NM_002589:exon1:c.G2752T:p.D918Y,PCDH7:NM_032456:exon1:c.G2752T:p.D918Y,PCDH7:NM_032457:exon1:c.G2752T:p.D918Y |
| 5 | 5303440 | 5303440 | G | A | ADAMTS16 | 36 | 137 | 0.2627737 | nonsynonymous SNV | ADAMTS16:NM_139056:exon19:c.G2849A:p.R950H |
| 5 | 34929953 | 34929953 | T | G | DNAJC21 | 6 | 90 | 0.0666667 | nonsynonymous SNV | DNAJC21:NM_001012339:exon1:c.T29G:p.V10G,DNAJC21:NM_001348420:exon1:c.T29G:p.V10G,DNAJC21:NM_194283:exon1:c.T29G:p.V10G |
| 5 | 54518188 | 54518188 | T | G | MCIDAS | 9 | 121 | 0.0743802 | nonsynonymous SNV | MCIDAS:NM_001190787:exon5:c.A422C:p.D141A |
| 5 | 74633127 | 74633127 | T | G | HMGCR | 14 | 119 | 0.1176471 | splicing | NM_000859:exon1:UTR5;NM_001130996:exon1:UTR5 |
| 5 | 131281176 | 131281176 | G | T | MEIKIN | 88 | 139 | 0.6330935 | nonsynonymous SNV | MEIKIN:NM_001303622:exon1:c.C23A:p.T8N |
| 5 | 133480554 | 133480554 | C | T | TCF7 | 36 | 143 | 0.2517483 | nonsynonymous SNV | TCF7:NM_201634:exon9:c.C797T:p.P266L |
| 5 | 167881029 | 167881029 | T | G | WWC1 | 9 | 123 | 0.0731707 | nonsynonymous SNV | WWC1:NM_001161661:exon18:c.T2582G:p.V861G,WWC1:NM_001161662:exon18:c.T2582G:p.V861G,WWC1:NM_015238:exon18:c.T2582G:p.V861G |
| 5 | 180651243 | 180651243 | T | G | TRIM41 | 13 | 142 | 0.0915493 | nonsynonymous SNV | TRIM41:NM_033549:exon1:c.T244G:p.W82G,TRIM41:NM_201627:exon1:c.T244G:p.W82G |
| 6 | 30918181 | 30918181 | T | G | DPCR1 | 7 | 61 | 0.1147541 | nonsynonymous SNV | DPCR1:NM_080870:exon2:c.T1940G:p.M647R |
| 6 | 30995852 | 30995852 | A | G | MUC22 | 12 | 80 | 0.15 | nonsynonymous SNV | MUC22:NM_001198815:exon3:c.A2644G:p.S882G,MUC22:NM_001318484:exon3:c.A2653G:p.S885G,MUC22:NM_001322469:exon3:c.A2653G:p.S885G |
| 6 | 35995995 | 35995995 | C | G | MAPK14 | 58 | 112 | 0.5178571 | nonsynonymous SNV | MAPK14:NM_001315:exon1:c.C61G:p.P21A,MAPK14:NM_139012:exon1:c.C61G:p.P21A,MAPK14:NM_139013:exon1:c.C61G:p.P21A,MAPK14:NM_139014:exon1:c.C61G:p.P21A |
| 6 | 55216140 | 55216140 | G | A | GFRAL | 20 | 48 | 0.4166667 | nonsynonymous SNV | GFRAL:NM_207410:exon5:c.G460A:p.A154T |
| 7 | 64438858 | 64438858 | C | T | ERV3-1-ZNF117;ZNF117 | 9 | 69 | 0.1304348 | nonsynonymous SNV | ERV3-1-ZNF117:NM_001348050:exon4:c.G1091A:p.G364E,ZNF117:NM_015852:exon4:c.G1091A:p.G364E |
| 7 | 73011743 | 73011743 | T | G | MLXIPL | 18 | 107 | 0.1682243 | nonsynonymous SNV | MLXIPL:NM_032951:exon9:c.A1372C:p.T458P,MLXIPL:NM_032952:exon9:c.A1372C:p.T458P,MLXIPL:NM_032953:exon9:c.A1372C:p.T458P,MLXIPL:NM_032954:exon9:c.A1372C:p.T458P |
| 7 | 100683179 | 100683179 | G | A | MUC17 | 11 | 94 | 0.1170213 | nonsynonymous SNV | MUC17:NM_001040105:exon3:c.G8482A:p.G2828S |
| 7 | 100684142 | 100684142 | T | G | MUC17 | 6 | 80 | 0.075 | nonsynonymous SNV | MUC17:NM_001040105:exon3:c.T9445G:p.S3149A |
| 7 | 100684864 | 100684864 | A | G | MUC17 | 8 | 94 | 0.0851064 | nonsynonymous SNV | MUC17:NM_001040105:exon3:c.A10167G:p.I3389M |
| 7 | 100684932 | 100684932 | T | C | MUC17 | 8 | 76 | 0.1052632 | nonsynonymous SNV | MUC17:NM_001040105:exon3:c.T10235C:p.V3412A |
| 7 | 150554504 | 150554504 | G | A | AOC1 | 52 | 92 | 0.5652174 | nonsynonymous SNV | AOC1:NM_001091:exon2:c.G946A:p.A316T,AOC1:NM_001272072:exon2:c.G946A:p.A316T |
| 8 | 10466031 | 10466031 | A | C | RP1L1 | 14 | 109 | 0.1284404 | nonsynonymous SNV | RP1L1:NM_178857:exon4:c.T5577G:p.D1859E |
| 8 | 144998902 | 144998902 | A | C | PLEC | 10 | 132 | 0.0757576 | nonsynonymous SNV | PLEC:NM_201378:exon31:c.T5153G:p.L1718R,PLEC:NM_201379:exon31:c.T5129G:p.L1710R,PLEC:NM_201380:exon31:c.T5606G:p.L1869R,PLEC:NM_201381:exon31:c.T5099G:p.L1700R,PLEC:NM_201382:exon31:c.T5195G:p.L1732R,PLEC:NM_201383:exon31:c.T5207G:p.L1736R,PLEC:NM_201384:exon31:c.T5195G:p.L1732R,PLEC:NM_000445:exon32:c.T5276G:p.L1759R |
| 9 | 2060892 | 2060892 | C | T | SMARCA2 | 31 | 69 | 0.4492754 | nonsynonymous SNV | SMARCA2:NM_001289396:exon9:c.C1598T:p.T533I,SMARCA2:NM_001289397:exon9:c.C1598T:p.T533I,SMARCA2:NM_003070:exon9:c.C1598T:p.T533I,SMARCA2:NM_139045:exon9:c.C1598T:p.T533I |
| 9 | 136213393 | 136213393 | A | C | MED22 | 16 | 89 | 0.1797753 | splicing | NM_133640:exon2:c.123+2T>G;NM_181491:exon2:c.123+2T>G |
| 9 | 139886942 | 139886942 | A | G | C9orf142 | 7 | 115 | 0.0608696 | nonsynonymous SNV | C9orf142:NM_001329678:exon1:c.A47G:p.E16G,C9orf142:NM_183241:exon1:c.A47G:p.E16G |
| 10 | 47682899 | 47682899 | G | A | ANTXRL | 28 | 65 | 0.4307692 | nonsynonymous SNV | ANTXRL:NM_001278688:exon15:c.G1327A:p.E443K |
| 10 | 103901137 | 103901137 | A | C | PPRC1 | 10 | 98 | 0.1020408 | nonsynonymous SNV | PPRC1:NM_001288727:exon5:c.A2872C:p.T958P,PPRC1:NM_001288728:exon5:c.A2512C:p.T838P,PPRC1:NM_015062:exon5:c.A2872C:p.T958P |
| 11 | 1272821 | 1272821 | G | A | MUC5B | 46 | 112 | 0.4107143 | nonsynonymous SNV | MUC5B:NM_002458:exon31:c.G14711A:p.R4904H |
| 11 | 74208345 | 74208345 | A | G | LOC100287896 | 7 | 58 | 0.1206897 | nonsynonymous SNV | LOC100287896:NM_001319240:exon2:c.A395G:p.N132S |
| 12 | 40883647 | 40883647 | T | C | MUC19 | 7 | 67 | 0.1044776 | unknown | UNKNOWN |
| 12 | 96389578 | 96389578 | C | G | HAL | 46 | 79 | 0.5822785 | nonsynonymous SNV | HAL:NM_001258334:exon2:c.G111C:p.K37N,HAL:NM_002108:exon2:c.G111C:p.K37N |
| 13 | 45150014 | 45150014 | G | C | TSC22D1 | 39 | 86 | 0.4534884 | nonsynonymous SNV | TSC22D1:NM_001243799:exon1:c.C197G:p.P66R,TSC22D1:NM_183422:exon1:c.C197G:p.P66R |
| 13 | 46170811 | 46170811 | A | C | ERICH6B | 12 | 84 | 0.1428571 | nonsynonymous SNV | ERICH6B:NM_182542:exon3:c.T330G:p.I110M |
| 13 | 51855226 | 51855226 | C | T | FAM124A | 47 | 81 | 0.5802469 | nonsynonymous SNV | FAM124A:NM_001242312:exon4:c.C1475T:p.A492V,FAM124A:NM_145019:exon5:c.C1583T:p.A528V |
| 14 | 25100314 | 25100314 | A | G | GZMB | 32 | 81 | 0.3950617 | nonsynonymous SNV | GZMB:NM_001346011:exon5:c.T671C:p.F224S,GZMB:NM_004131:exon5:c.T707C:p.F236S |
| 14 | 88945745 | 88945745 | A | C | PTPN21 | 5 | 83 | 0.060241 | nonsynonymous SNV | PTPN21:NM_007039:exon13:c.T2030G:p.V677G |
| 14 | 105412225 | 105412225 | T | G | AHNAK2 | 5 | 33 | 0.1515152 | nonsynonymous SNV | AHNAK2:NM_001350929:exon7:c.A9263C:p.N3088T,AHNAK2:NM_138420:exon7:c.A9563C:p.N3188T |
| 14 | 105414701 | 105414701 | T | C | AHNAK2 | 10 | 81 | 0.1234568 | nonsynonymous SNV | AHNAK2:NM_001350929:exon7:c.A6787G:p.T2263A,AHNAK2:NM_138420:exon7:c.A7087G:p.T2363A |
| 15 | 23685712 | 23685712 | T | C | GOLGA6L2 | 9 | 70 | 0.1285714 | nonsynonymous SNV | GOLGA6L2:NM_001304388:exon8:c.A1910G:p.E637G |
| 15 | 24923062 | 24923062 | A | G | NPAP1 | 8 | 72 | 0.1111111 | nonsynonymous SNV | NPAP1:NM_018958:exon1:c.A2048G:p.N683S |
| 15 | 89417205 | 89417205 | G | A | ACAN | 53 | 93 | 0.5698925 | nonsynonymous SNV | ACAN:NM_013227:exon17:c.G7466A:p.R2489Q |
| 16 | 14014231 | 14014231 | T | G | ERCC4 | 9 | 102 | 0.0882353 | splicing | NM_005236:exon1:c.207+2T>G |
| 16 | 30016615 | 30016615 | T | G | INO80E | 10 | 97 | 0.1030928 | nonsynonymous SNV | INO80E:NM_001304562:exon6:c.T470G:p.V157G,INO80E:NM_173618:exon7:c.T587G:p.V196G |
| 16 | 65016048 | 65016048 | G | A | CDH11 | 22 | 49 | 0.4489796 | nonsynonymous SNV | CDH11:NM_001330576:exon7:c.C778T:p.P260S,CDH11:NM_001308392:exon8:c.C1156T:p.P386S,CDH11:NM_001797:exon8:c.C1156T:p.P386S |
| 16 | 86545095 | 86545095 | A | C | FOXF1 | 14 | 117 | 0.1196581 | nonsynonymous SNV | FOXF1:NM_001451:exon1:c.A920C:p.H307P |
| 17 | 4619841 | 4619841 | A | C | ARRB2 | 7 | 70 | 0.1 | nonsynonymous SNV | ARRB2:NM_001257331:exon4:c.A250C:p.T84P,ARRB2:NM_199004:exon4:c.A250C:p.T84P,ARRB2:NM_001257328:exon5:c.A295C:p.T99P,ARRB2:NM_001257329:exon5:c.A295C:p.T99P,ARRB2:NM_001257330:exon5:c.A295C:p.T99P,ARRB2:NM_004313:exon5:c.A295C:p.T99P |
| 17 | 37762617 | 37762617 | A | C | NEUROD2 | 17 | 162 | 0.1049383 | nonsynonymous SNV | NEUROD2:NM_006160:exon2:c.T236G:p.L79R |
| 17 | 40359729 | 40359729 | T | G | STAT5B | 8 | 107 | 0.0747664 | nonsynonymous SNV | STAT5B:NM_012448:exon16:c.A1924C:p.N642H |
| 17 | 56291211 | 56291211 | C | A | MKS1 | 5 | 125 | 0.04 | stopgain | MKS1:NM_001321268:exon6:c.G55T:p.E19X,MKS1:NM_001165927:exon7:c.G634T:p.E212X,MKS1:NM_001321269:exon7:c.G664T:p.E222X,MKS1:NM_001330397:exon7:c.G664T:p.E222X,MKS1:NM_017777:exon7:c.G664T:p.E222X |
| 17 | 62657945 | 62657945 | A | C | SMURF2 | 9 | 157 | 0.0573248 | splicing | NM_022739:exon1:c.52+2T>G |
| 17 | 70119716 | 70119716 | A | C | SOX9 | 13 | 128 | 0.1015625 | nonsynonymous SNV | SOX9:NM_000346:exon3:c.A718C:p.T240P |
| 17 | 77755917 | 77755917 | A | G | CBX2 | 6 | 156 | 0.0384615 | nonsynonymous SNV | CBX2:NM_032647:exon4:c.A605G:p.D202G |
| 18 | 28586896 | 28586896 | A | C | DSC3 | 11 | 65 | 0.1692308 | nonsynonymous SNV | DSC3:NM_001941:exon12:c.T1865G:p.L622R,DSC3:NM_024423:exon12:c.T1865G:p.L622R |
| 19 | 8175940 | 8175940 | A | C | FBN3 | 6 | 106 | 0.0566038 | splicing | NM_032447:exon33:c.4210+2T>G;NM_001321431:exon33:c.4210+2T>G |
| 19 | 21300738 | 21300738 | T | C | ZNF714 | 14 | 80 | 0.175 | nonsynonymous SNV | ZNF714:NM_182515:exon5:c.T1268C:p.L423P |
| 19 | 21366245 | 21366245 | G | C | ZNF431 | 6 | 54 | 0.1111111 | nonsynonymous SNV | ZNF431:NM_001319124:exon5:c.G1142C:p.G381A,ZNF431:NM_133473:exon5:c.G1139C:p.G380A,ZNF431:NM_001319126:exon6:c.G866C:p.G289A,ZNF431:NM_001319127:exon6:c.G818C:p.G273A |
| 19 | 22156863 | 22156863 | C | A | ZNF208 | 8 | 50 | 0.16 | nonsynonymous SNV | ZNF208:NM_007153:exon4:c.G973T:p.V325F |
| 19 | 22362945 | 22362945 | A | C | ZNF676 | 8 | 57 | 0.1403509 | nonsynonymous SNV | ZNF676:NM_001001411:exon3:c.T1574G:p.I525R |
| 19 | 22940877 | 22940877 | G | T | ZNF99 | 6 | 66 | 0.0909091 | nonsynonymous SNV | ZNF99:NM_001080409:exon4:c.C1834A:p.Q612K |
| 19 | 23040712 | 23040712 | T | C | ZNF723 | 12 | 72 | 0.1666667 | nonsynonymous SNV | ZNF723:NM_001349726:exon4:c.T1019C:p.L340P |
| 19 | 23329203 | 23329203 | G | A | ZNF730 | 4 | 64 | 0.0625 | nonsynonymous SNV | ZNF730:NM_001277403:exon4:c.G1357A:p.E453K |
| 19 | 23926616 | 23926616 | C | G | ZNF681 | 5 | 51 | 0.0980392 | nonsynonymous SNV | ZNF681:NM_138286:exon4:c.G1736C:p.R579T |
| 19 | 39914468 | 39914468 | G | T | PLEKHG2 | 58 | 100 | 0.58 | nonsynonymous SNV | PLEKHG2:NM_001351693:exon19:c.G2518T:p.V840F,PLEKHG2:NM_022835:exon19:c.G2695T:p.V899F |
| 19 | 44117798 | 44117798 | T | C | SRRM5 | 13 | 79 | 0.164557 | nonsynonymous SNV | SRRM5:NM_001145641:exon1:c.T1525C:p.C509R |
| 19 | 48182845 | 48182845 | G | A | BICRA | 65 | 112 | 0.5803571 | nonsynonymous SNV | BICRA:NM_015711:exon6:c.G418A:p.G140R |
| 19 | 52222842 | 52222842 | C | G | HAS1 | 58 | 103 | 0.5631068 | nonsynonymous SNV | HAS1:NM_001297436:exon2:c.G316C:p.D106H,HAS1:NM_001523:exon2:c.G319C:p.D107H |
| 19 | 53410536 | 53410536 | C | G | ZNF888 | 6 | 79 | 0.0759494 | nonsynonymous SNV | ZNF888:NM_001310127:exon3:c.G1039C:p.V347L |
| 19 | 53856405 | 53856405 | G | C | ZNF845 | 7 | 73 | 0.0958904 | nonsynonymous SNV | ZNF845:NM_138374:exon4:c.G2477C:p.S826T,ZNF845:NM_001321522:exon5:c.G2477C:p.S826T,ZNF845:NM_001321523:exon5:c.G2477C:p.S826T,ZNF845:NM_001321524:exon5:c.G2477C:p.S826T |
| 19 | 56200661 | 56200661 | A | C | EPN1 | 14 | 107 | 0.1308411 | splicing | NM_001321263:exon5:c.604-2A>C;NM_001130072:exon5:c.604-2A>C |
| 19 | 57133670 | 57133670 | T | G | ZNF71 | 6 | 90 | 0.0666667 | nonsynonymous SNV | ZNF71:NM_021216:exon3:c.T1015G:p.S339A |
| 19 | 58290497 | 58290497 | T | G | ZNF586 | 7 | 81 | 0.0864198 | nonsynonymous SNV | ZNF586:NM_001077426:exon2:c.T415G:p.L139V,ZNF586:NM_017652:exon3:c.T542G:p.I181S,ZNF586:NM_001204814:exon4:c.T413G:p.I138S |
| 19 | 58549317 | 58549317 | G | A | ZSCAN1 | 41 | 99 | 0.4141414 | nonsynonymous SNV | ZSCAN1:NM_182572:exon3:c.G113A:p.R38H |
| 21 | 36042010 | 36042010 | T | G | CLIC6 | 13 | 110 | 0.1181818 | nonsynonymous SNV | CLIC6:NM_001317009:exon1:c.T323G:p.V108G,CLIC6:NM_053277:exon1:c.T323G:p.V108G |
| 21 | 36042144 | 36042144 | T | G | CLIC6 | 13 | 112 | 0.1160714 | nonsynonymous SNV | CLIC6:NM_001317009:exon1:c.T457G:p.S153A,CLIC6:NM_053277:exon1:c.T457G:p.S153A |
| 22 | 40082235 | 40082235 | A | C | CACNA1I | 13 | 129 | 0.1007752 | nonsynonymous SNV | CACNA1I:NM_001003406:exon36:c.A6392C:p.H2131P,CACNA1I:NM_021096:exon37:c.A6497C:p.H2166P |
| 22 | 45792305 | 45792305 | T | A | SMC1B | 18 | 40 | 0.45 | nonsynonymous SNV | SMC1B:NM_001291501:exon7:c.A1175T:p.Q392L,SMC1B:NM_148674:exon7:c.A1175T:p.Q392L |
| X | 34962206 | 34962206 | G | A | FAM47B | 29 | 36 | 0.8055556 | nonsynonymous SNV | FAM47B:NM_152631:exon1:c.G1258A:p.V420M |
| X | 100382575 | 100382575 | G | A | CENPI | 34 | 34 | 1 | nonsynonymous SNV | CENPI:NM_001318523:exon9:c.G995A:p.C332Y,CENPI:NM_006733:exon10:c.G995A:p.C332Y,CENPI:NM_001318521:exon11:c.G995A:p.C332Y |
| X | 100746193 | 100746193 | T | G | ARMCX4 | 10 | 44 | 0.2272727 | nonsynonymous SNV | ARMCX4:NM_001256155:exon2:c.T2617G:p.L873V |
| X | 114425862 | 114425862 | T | C | RBMXL3 | 6 | 29 | 0.2068966 | nonsynonymous SNV | RBMXL3:NM_001145346:exon1:c.T1858C:p.W620R |
| X | 114425988 | 114425988 | T | C | RBMXL3 | 6 | 26 | 0.2307692 | nonsynonymous SNV | RBMXL3:NM_001145346:exon1:c.T1984C:p.C662R |
| X | 124455313 | 124455313 | T | C | TEX13C | 7 | 45 | 0.1555556 | nonsynonymous SNV | TEX13C:NM_001195272:exon1:c.T1345C:p.C449R |

**Table S5.** Structural variants identified by whole genome sequencing.

| CHR  _A | START_A | END_A | CHR  _B | START_B | END_B | TYPE | left_nearest  _gene | right_nearest  _gene | distance |
| --- | --- | --- | --- | --- | --- | --- | --- | --- | --- |
| X | 4633351 | 4633372 | 13 | 109649664 | 109649665 | BND | AC074035.1 | MYO16 | -1 |
| 3 | 28282921 | 28282922 | 2 | 157176649 | 157176650 | BND | CMC1 | NR4A2 | -1 |
| 3 | 28283281 | 28283282 | 2 | 157176729 | 157176730 | BND | CMC1 | NR4A2 | -1 |
| 21 | 39931636 | 39931637 | 20 | 23984146 | 23984147 | BND | ERG | GGTLC1 | -1 |
| 20 | 23984146 | 23984147 | 21 | 39931636 | 39931637 | BND | GGTLC1 | ERG | -1 |
| 2 | 157176649 | 157176650 | 3 | 28282921 | 28282922 | BND | NR4A2 | CMC1 | -1 |
| 2 | 157176729 | 157176730 | 3 | 28283281 | 28283282 | BND | NR4A2 | CMC1 | -1 |
| 8 | 78290126 | 78290127 | 3 | 81341421 | 81341422 | BND | RP11-38H17.1 | RP11-520D19.2 | -1 |
| 3 | 81341421 | 81341422 | 8 | 78290126 | 78290127 | BND | RP11-520D19.2 | RP11-38H17.1 | -1 |
| 13 | 109649644 | 109649665 | X | 4633371 | 4633372 | BND | MYO16 | AC074035.1 | -1 |
| 10 | 72020081 | 72020084 | 10 | 72058470 | 72058473 | DEL | NPFFR1 | LRRC20 | 38389 |
| 11 | 112211130 | 112211134 | 11 | 121332875 | 121332879 | DEL | RP11-356J5.12 | SORL1 | 9121745 |
| 14 | 22907999 | 22908000 | 14 | 22918138 | 22918139 | DEL | AE000661.37 | AE000661.37 | 10139 |
| 14 | 22564893 | 22564894 | 14 | 22919071 | 22919072 | DEL | AE000660.1 | AE000661.37 | 354178 |
| 3 | 47104218 | 47104221 | 3 | 47109460 | 47109463 | DEL | SETD2 | SETD2 | 5242 |
| 7 | 38293017 | 38293018 | 7 | 38369945 | 38369946 | DEL | STARD3NL | RP11-121A8.1 | 76928 |
| 7 | 38309136 | 38309137 | 7 | 38393313 | 38393314 | DEL | STARD3NL | RP11-121A8.1 | 84177 |
| 20 | 14179096 | 14179099 | 20 | 23984171 | 23984174 | INV | MACROD2 | GGTLC1 | 9805075 |
|  |  |  |  |  |  |  |  |  |  |

We noted a double-hit of the *SETD2* gene (NM_014159), with a somatic single-nucleotide splicing variant (chr3:47205343:C>T)- 1 base downstream of exon1, as well as a somatic large deletion of ~5.2 kb (chr3:47104218-47109460) **(Figure S4A).** Both of these somatic alterations were validated by PCR **(Figure S4B).** The chimeric sequence from the deletion and splicing SNV were further validated by Sanger sequencing **(Figure S4C and S4D).** Whole transcriptome sequencing shows that the splicing SNV and genomic deletion affected the first intron **(Figure S4E)** and created a cryptic 3’ end transcription termination site **(Figure S4F),** respectively. This molecular profile is consistent with the genetics of HSTCL, where *SETD2* is commonly altered.

**Figure S4 (following page). Aberrant genomic and transcriptomic alterations in the *SETD2* gene in a single case of HSTCL.** A) Genomic locations of the splicing SNV and structural variation (exon13-deletion) within the coding domains of the *SETD2* gene (Transcript ID: NM_014159). B) Gel electrophoresis photograph of the gDNA PCR products flanking both the ~5.2kb deletion (left) and the splicing SNV (right); T: tumor, N: buccal swab as normal and 1 kb ladder in steps of 100 bp on the leftmost. C) Sanger sequencing of the chimeric gDNA sequence flanking the large deletion within *SETD2* gene. Red vertical line depicts the exact location within the sequence where the wildtype deleted sequence used to be. D) Somatic Sanger-based validation of the splicing SNV. Arrows point to the location of interrogation for the splicing SNV (top: Tumor, bottom: Buccal Swab as normal). E) IGV visualization of the STAR-aligned RNA-seq alignments in the genomic locality of the splicing SNV (hg19, chr3:47205343:C>T), 1 bp downstream of *SETD2’s* exon1, together with alignments spanning across the wild-type exon1-intron1 junction. F) IGV visualization of the STAR-aligned RNA-seq alignments downstream of exon12 showing the novel cryptic splicing junction as a consequence of the exon13-deletion.

A

B

C

F

E

D
